# Characteristics and quality of reporting qualitative nursing research related to the COVID-19 pandemic: a systematic search and critical review

**DOI:** 10.1186/s12912-024-02138-x

**Published:** 2024-07-22

**Authors:** Ian-In Vong, Monique Rothan-Tondeur, Rita Georges Nohra

**Affiliations:** 1 Nursing Sciences Research Chair, Laboratory Educations and Health Promotion (LEPS), University Sorbonne Paris Nord, UFR SMBH, Villetaneuse, EA 3412, F-93430 France; 2 Nursing Sciences Research Chair, Assistance Publique-Hôpitaux de Paris (AP-HP), Paris, F- 75005 France

**Keywords:** COVID-19, Nursing research, Qualitative research, Methodological quality, Critical review

## Abstract

**Background:**

The COVID-19 (Coronavirus disease of 2019) pandemic caused major disruption to nursing research, especially qualitative research. Researchers had to overcome numerous challenges that potentially impacted the quality of the studies carried out.

**Objectives:**

The aim of this study is to assess the characteristics and quality of reporting qualitative nursing articles on the COVID-19 pandemic.

**Methods:**

A systematic search and critical review using content analysis was conducted on published nurse-led articles using a qualitative approach related to the COVID-19 pandemic. A combination of the Consolidated Criteria for Reporting Qualitative Research (COREQ) and Standards for Reporting Qualitative Research (SRQR) checklists and additional items identified from the literature were used to assess the characteristics and overall quality of reporting of qualitative research.

**Results:**

Out of 63,494 articles screened, 444 met the inclusion criteria. Most studies were published in high-impact, Quartile 1 journals, with the majority originating from the USA. Common themes included workforce experiences and the impact of pandemic restrictions. Methodological quality varied, with a notable underuse of standardized reporting checklists. Despite pandemic-induced challenges in data collection, interviews remained the predominant method. However, the adoption of remote research methods and analysis software was limited.

**Discussion:**

The findings underscore the resilience and adaptability of nursing researchers during the pandemic. High-quality publications in top-tier journals indicate rigorous academic standards. However, the low utilization of reporting checklists suggests a need for greater emphasis on methodological transparency and adherence to established quality guidelines. This review highlights the importance of enhancing qualitative research practices to improve the rigor and reliability of studies, particularly in crisis contexts.

## Introduction

The COVID-19 pandemic has threatened the health and well-being of global citizens which has led to a significant change in the attitude, lifestyle, and behavior of people from diverse professions [1]. Nurses have been and remain central to the pandemic––nurses are central to preventative, curative and palliative activities associated with COVID-19, and have taken these roles on in addition to their usual roles [2]. Nurses reported low job satisfaction, high levels of burnout, stress, and anxiety [3]. Researchers have experienced a decline in research motivation [4]. Scientific productivity, particularly among female academics, has suffered due to increased childcare responsibilities and psychological distress [5]. And parent researchers struggled to balance work and family responsibilities during the pandemic [6].

On the other hand, the travel restrictions and lockdown during the pandemic have undoubtedly affected all aspects of research, including qualitative research [7, 8]. Qualitative nursing research is essential and important for understanding patient experiences, exploring complex healthcare phenomena, and guiding patient-centered care [9]. It provides insights into the subjective experiences, perceptions, and emotions of patients, families, and providers [10], bringing a holistic perspective to understanding the phenomena under study [11]. With qualitative methodologies, insight can be gained regarding the social responses to this pandemic, they are also the best methods to help explain, address, and plan for emergencies and pandemics, such as COVID-19 [7, 12, 13]. Restrictions during the pandemics made traditional data collection methods challenging [1, 14]. Nurse researchers had to adapt to perform data collection in a virtual environment, shifting from face-to-face interviews to telephone or online meetings [1]; research participants were unwilling to show their faces at virtual meetings, and face-to-face interviews were only allowed with masks on [7, 8]. These changes affected the quality and richness of data collection, missing important non-verbal elements such as attitude, gesture, and context [15, 16].

Given the disruptive impact of the COVID-19 pandemic on nursing qualitative research activities, and deleterious effects on nurses, like emotional exhaustion [17], psychological distress [18], and burnout [3, 19], but nurse researchers have also been very responsive to the pandemic, the *Journal of Advanced Nursing* has received hundreds of manuscripts focused on the pandemic, and more than 200 papers published on the COVID-19 pandemic in 2 years [2]. We doubted the quality of the publication. Scholarly journals are the most important media source for the dissemination of such research findings and information related to connecting this new evidence to practice [20] and nursing publication plays an essential role in improving nurses’ knowledge of new information and interesting this knowledge into nursing practice [21]. Together these phenomena might run the risk of producing poor quality qualitative research. Current literature provides two bibliometric analyses of COVID-19 research published in nursing journal, these provide the readers with only objective information on nursing publication related to COVID-19. The existing literature lacks comprehensive reviews that specifically focus on the characteristics and reporting quality of qualitative nursing research related to COVID-19. This study addresses this gap by providing a thorough analysis, which is crucial for guiding future research efforts and improving the overall quality of qualitative studies in nursing. By emphasizing the importance of maintaining high research quality, this study aims to contribute valuable insights that can inform future research, policymaking, and practice in nursing.

Providing a critical review of COVID-19 qualitative nursing research is an unmet need. To achieve this goal, we designed a systematic literature search including all available COVID-19 nursing qualitative articles using a large task force dedicated to the analysis of high-volume articles. We aimed at investigating the characteristics and the methodological quality assessment of reporting COVID-19 qualitative nursing publications.

## Methods

We conducted a systematic literature search and a critical review using content analysis. This type of content analysis was to enable the production of measurements, occurrences, or comparisons through statistical or quantitative methods [22]. This review builds upon the methods utilized in two similar reviews [23, 24], which assessed the characteristics of articles and described the methodological quality of the articles by presenting the percentage of compliance with each item of a standardized methodological reporting quality checklist. Our study adopted a pre-established checklist which was designed based on the Consolidated Criteria for Reporting Qualitative Research (COREQ) [25] and the Standards for Reporting Qualitative Research (SRQR) [26], along with other items identified in the literature to examine the quality of reporting in qualitative research.

This study is an ancillary study that extracted articles related to COVID-19 from the database of a large study aims to assess the characteristics and reporting quality using a qualitative approach in the field of nursing from 2012 to January 2023.

### Search strategy

Several databases were consulted to ensure the inclusion of relevant studies in the field of nursing. The main databases are academic and medical databases, such as PubMed, CINAHL (Cumulative Index to Nursing and Allied Health Literature), Cairn, Embase, Web of science and Scopus. Document search strategies are developed using the MeSH thesaurus (Medical subject headings) and related keywords. The MEDLINE strategy has been developed and tested by the research team: “nursing research“[MH] OR “nursing research“[TW] OR (“nursing research“[Title/Abstract:~2]) OR nurs*[affiliation]) AND (“qualitative research“[MH] OR “qualitative research“[TIAB] OR “qualitative study“[TIAB] OR “qualitative studies“[TIAB] OR “grounded theory“[TIAB] OR “phenomenology“[TIAB] OR “ethnography“[TIAB] OR (“qualitative study“[Title/Abstract:~2] OR “qualitative studies“[Title/Abstract:~2] OR “qualitative research“[Title/Abstract:~2] OR “qualitative theory“[Title/Abstract:~2] OR “qualitative theories“[Title/Abstract:~2] OR “grounded study“[Title/Abstract:~2] OR “grounded studies“[Title/Abstract:~2] OR “grounded theory“[Title/Abstract:~2] OR “grounded theories“[Title/Abstract:~2] OR “grounded research“[Title/Abstract:~2] OR “ethnological study“[Title/Abstract:~2] OR “ethnological studies“[Title/Abstract:~2] OR “ethnological theory“[Title/Abstract:~2] OR “ethnological theories“[Title/Abstract:~2] OR “ethnological research“[Title/Abstract:~2] OR “phenomenological study“[Title/Abstract:~2] OR “phenomenological studies“[Title/Abstract:~2] OR “phenomenological theory“[Title/Abstract:~2] OR “phenomenological theories“[Title/Abstract:~2] OR “phenomenological research“[Title/Abstract:~2]. Then, a hand search was conducted to identified articles related to COVID-19. The literature search was performed between June 2023 to August 2023.

### Inclusion and exclusion criteria

Any qualitative nursing research related to COVID-19 was included. The first authors must be nurses. The language was limited to English and French. Both peer-reviewed and pre-prints articles were included.

Articles related to non-human samples and full-text unavailable were excluded.

### Article screening

We followed the PRISMA 2020 (Preferred Reporting Items for Systematic Reviews and Meta-Analyses) for article selection. All articles yielded through an initial search from the databases were exported into Rayyan Software, a web-based tool designed to conduct and coordinate systematic literature reviews. Hand search was performed to identify articles related to COVID-19, and duplicates were removed. Next, affiliations were examined to determine if the first author was a nurse, and then titles and abstracts were reviewed to determine if the publication met inclusion and exclusion criteria. Two researchers finished the screening independently. Any discrepant result was discussed by the two reviewers and resolved by consensus, or where necessary, a third researcher was involved. Finally, the articles that met the inclusion and exclusion criteria were selected for full-text reading.

### Data extraction and data analysis

We used the pre-established checklist combining items from the SRQR and COREQ checklists and adding other items identified in the literature to answer the objective of this study. The checklist included 33 items seen in Tables 1 and 9 items regarding characteristics of the articles, and 24 items regarding methodological quality assessment. The checklist was pilot-tested and revised. Revisions were made after discussion among the researchers and included clarification of checklist items and the response of researchers to each item. For items of the characteristics of the articles, data were extracted to Excel (Excel 2020, Microsoft Excel, Redmond, WA, USA) for categorization. For items of methodological quality assessment, ATLAS.ti software (version 23.2.1) was used. All identified articles were imported into the software for content analysis with the use of a coding function, codes were created according to the items on the data extraction checklist, researcher read the content of the full-text articles one by one, then identified and coded the phrases according to the codes. For example, the code “field note” was created, and the researcher identified and coded the content if it is mentioned in the article. The frequency of each code was calculated to identify the methodological quality of the included articles.

**Table 1:** Data extraction checklist

Data extraction checklist	
Characteristics of the COVID-19 Qualitative Nursing Research	
Data related to the journals	Journals
	Impact factor
	Quartile
Data related to the articles	Year of publication
	Country
Characteristics of researchers	Education level
	Affiliations
Focuses and Populations of the articles	Focuses
	Populations
**Methodological Quality Assessment of COVID-19 Qualitative Nursing Research**	
Methodological Orientation	Approach adopted specified
	Reporting quality checklist mentioned
Data Collection	Method of data collection mentioned
	Date of data collection mentioned
	Interviewer mentioned
	Audio/Visual recording mentioned
	Field note mentioned
	Interview guideline mentioned
	Interview guideline pre-tested
Participants	Sample size mentioned
	Sampling methods mentioned
	Description of sample
Data analysis	Method of data analysis mentioned
	Investigator triangulation mentioned
	Data triangulation mentioned
	Ecological triangulation mentioned
	Source triangulation mentioned
	Using software
	Data saturation mentioned
Presentation of results	Narrative presentation
	Quotation presented
	Recurrence of codes presented
	Presentation of graphs
	Participants checking report mentioned

### Ethical considerations

This study is a review based on published articles; ethical approval was not required.

## Results

**Fig. 1:**
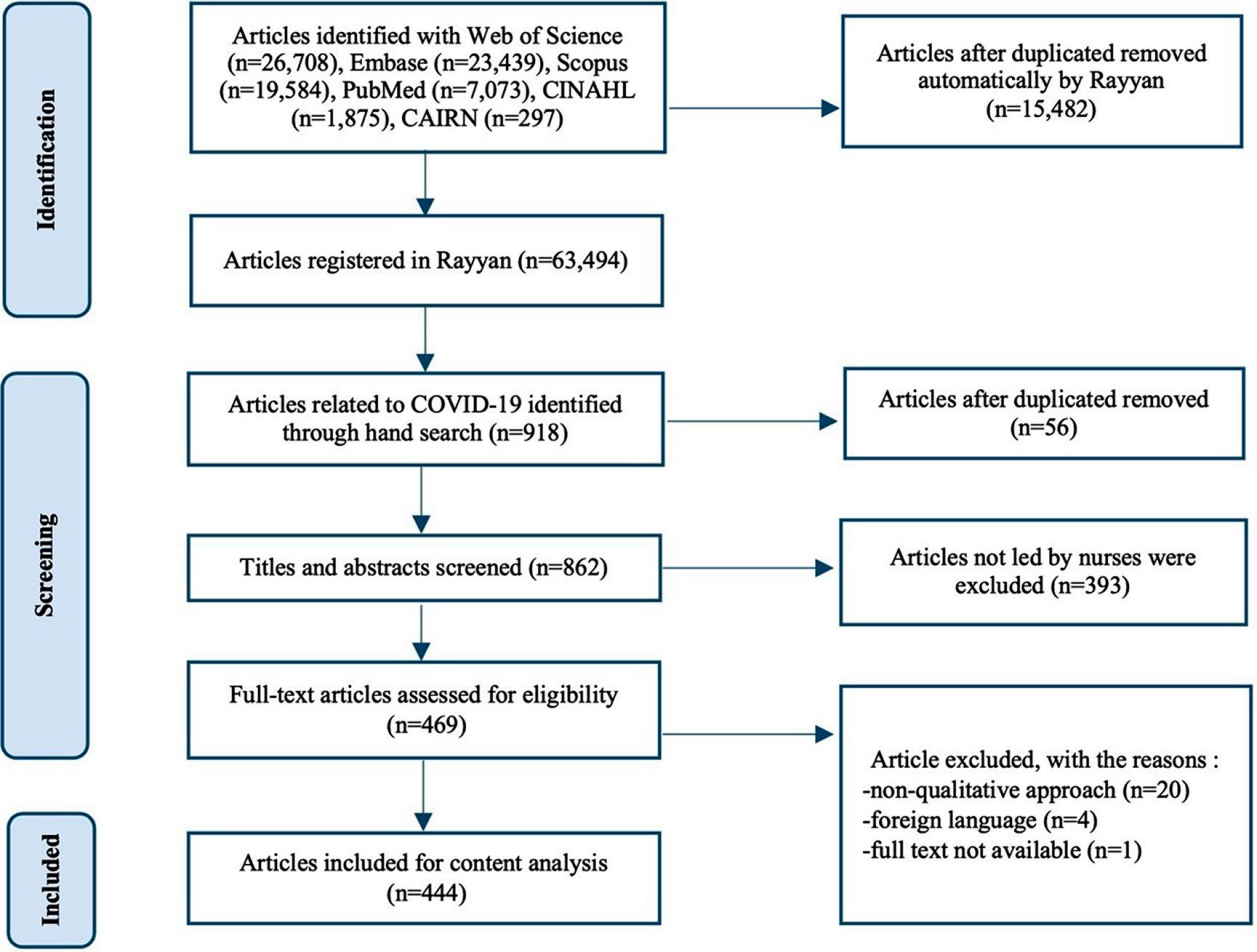
Flowchart of articles screening

A total of 63,494 articles were registered in Rayyan software. Of these, 918 articles (1.44%) were related to COVID-19. After the exclusion of 56 articles due to duplication, the titles, and abstracts of all the articles were examined and 393 articles were excluded due to affiliations in which the first author was not a nurse. The remaining studies were reviewed in full-text. There were 20 articles excluded due to articles with a non-qualitative approach, 4 articles written in a foreign language were excluded, and 1 article was excluded because of full-texted unavailable. A total of 444 full-text articles related to COVID-19 were analyzed. The flowchart is presented in Fig. 1.

### Characteristics of the COVID-19 qualitative nursing research

The 444 included articles were published in 196 different journals, one of which was published on MedRxiv, an online pre-print platform for non-peer-reviewed research, with the most articles being published in the *International Journal of Environmental Research and Public Health* (*n* = 28, 14.3%).

Table 2 shows the most productive journals in terms of COVID-19 nursing qualitative publications. With regard to the quartile of the journals, the studies were published most frequently in Q1 journals (*n* = 260, 58.6%), followed by Q2 (*n* = 118, 26.6%), Q3 (*n* = 49, 11.0%), Q4 (*n* = 12, 2.7%). The impact factors for each journal are grouped into 6 categories: Of the 444 articles, impact factor below 1 (*n* = 54, 12.2%), impact factor between 1 and 1.999 (*n* = 72, 16.2%). In addition, impact factor between 2 and 2.999 (*n* = 107, 24.1%), impact factor between 3 and 3.999 (*n* = 92, 20.7%), impact factor between 4 and 4.999 (*n* = 87, 19.6%), and impact factor of 5 or higher (*n* = 29, 6.5%). And 3 articles published in journals with an impact factor which is not applicable.

**Table 2:** Top 25 most productive nursing journals

Top 25 most productive nursing journals		
Journal	Number	%
International Journal of Environmental Research and Public Health	28	14.3
Journal of Nursing Management	21	10.7
Journal of Advanced Nursing	15	7.7
BMJ Open	12	6.1
Journal of Clinical Nursing	11	5.6
Open Access Macedonian Journal of Medical Sciences	11	5.6
Frontiers in Public Health	9	4.6
BMC Public Health	8	4.1
BMC Nursing	7	3.6
International Nursing Review	7	3.6
Nursing Ethics	6	3.1
PLOS ONE	6	3.1
Iranian Journal of Nursing and Midwifery Research	5	2.6
Journal of Pediatric Nursing	5	2.6
Midwifery	5	2.6
Nurse Education Today	5	2.6
BMC Health Services Research	4	2.0
International Journal of Qualitative Studies on Health and Well-being	4	2.0
Journal of Professional Nursing	4	2.0
Nursing in Critical Care	4	2.0
Nursing Forum	4	2.0
Nursing Outlook	4	2.0
Nurse Education in Practice	4	2.0
Public Health Nursing	4	2.0
Women and Birth	4	2.0
Total number of journals: 196		

**Fig. 2:**
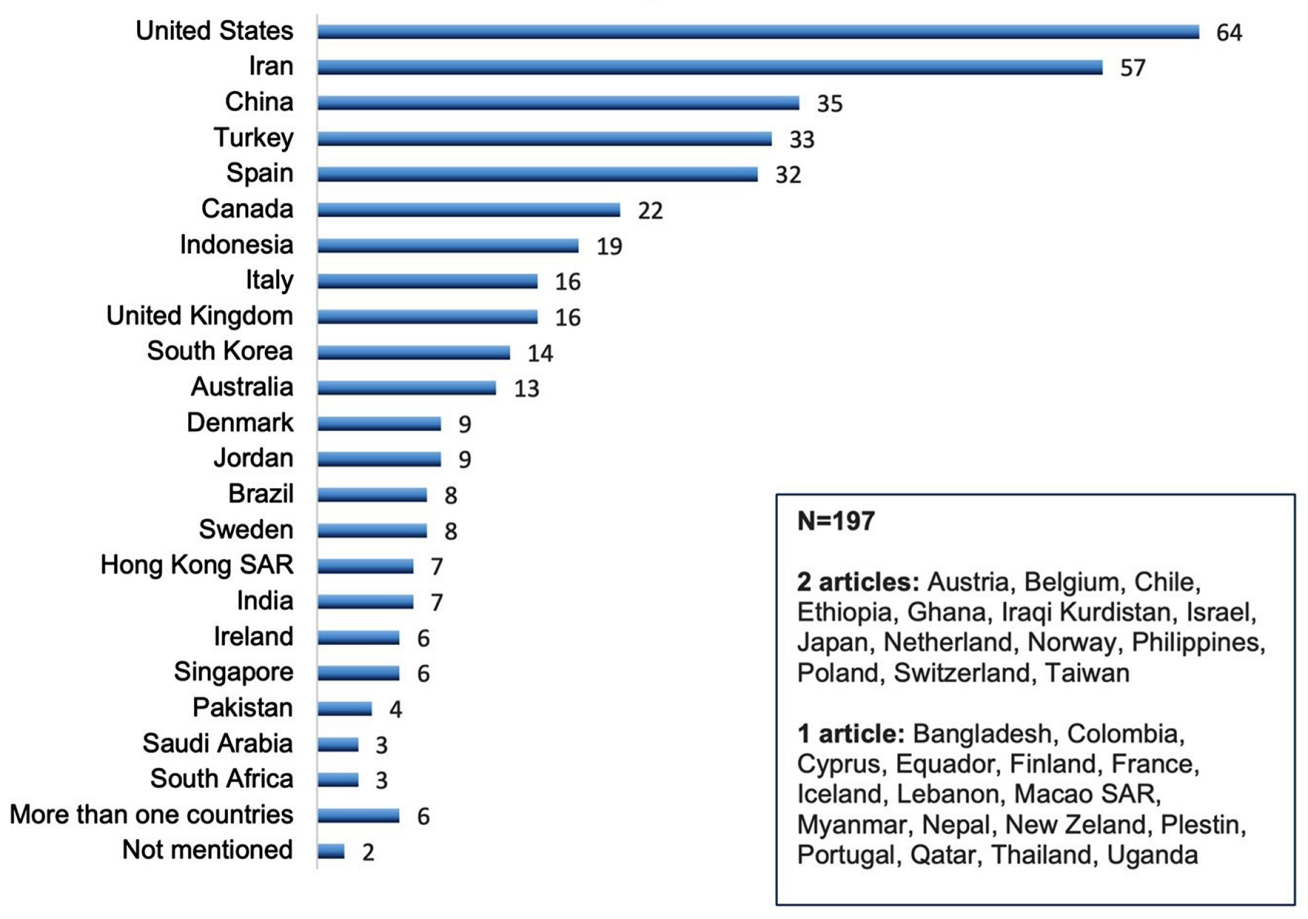
Distribution of countries of COVID-19 related qualitative nursing research published

We then assessed the distribution of countries among all the included publications. The top 10 publishing countries were the United States (*n* = 64, 14.4%), Iran (*n* = 57, 12.8%), China (*n* = 35, 7.9%), Turkey (*n* = 33, 7.4%), Spain (*n* = 32, 7.2%), Canada (*n* = 22, 5.0%), Indonesia (*n* = 19, 4.3%), Italy (*n* = 16, 3.6%) and the United Kingdom (*n* = 16, 3.6%) respectively, and South Korea (*n* = 14, 3.2%), see Fig. 2. Regarding the year of publication, 27 articles (6.1%) were published in 2020, 170 articles (38.3%) in 2021, and 240 articles (54.1%) in 2022.

The academic qualifications of the first authors were reported in 150 (33.8%) of the 444 articles. Of these, 113 (75.3%) first authors have a Ph.D degree (*n* = 113, 75.3%), Ph.D. candidates (*n* = 3, 2.0%), Ph.D. students (*n* = 7, 4.7%), Master degree (*n* = 23, 15.3%), Master students (*n* = 2, 1.3%), and Bachelor degree (*n* = 2, 1.3%). The affiliations of the first author were the universities (*n* = 395, 89.7%), the hospitals (*n* = 34, 7.7%), research centers (*n* = 12, 2.7%), and independent researchers (*n* = 1, 0.2%).

**Fig. 3:**
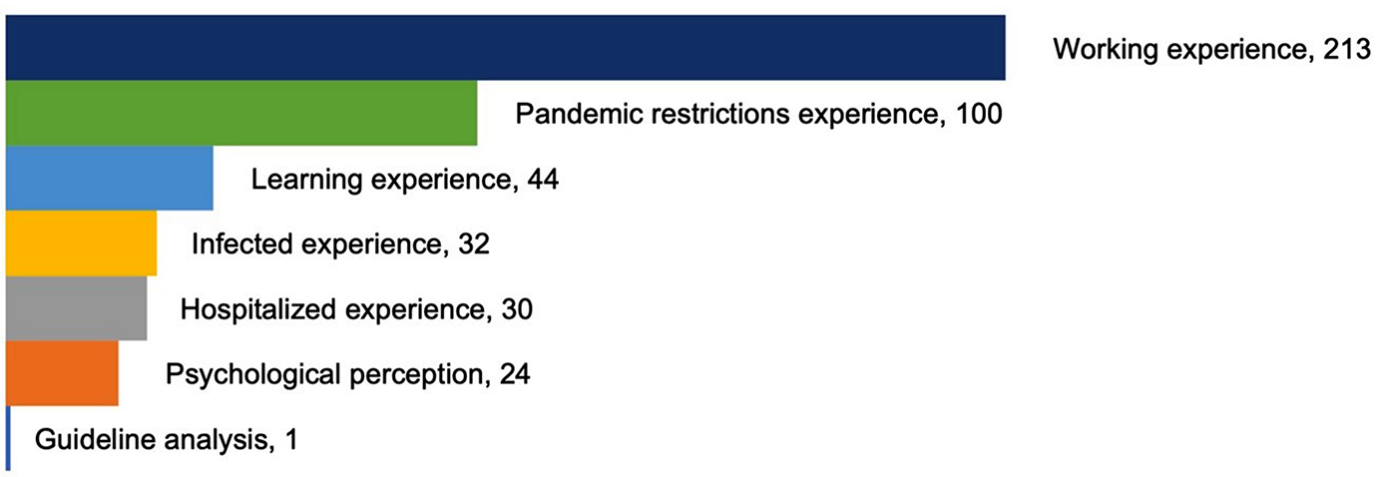
Focuses of COVID-19 qualitative nursing research

**Fig. 4:**
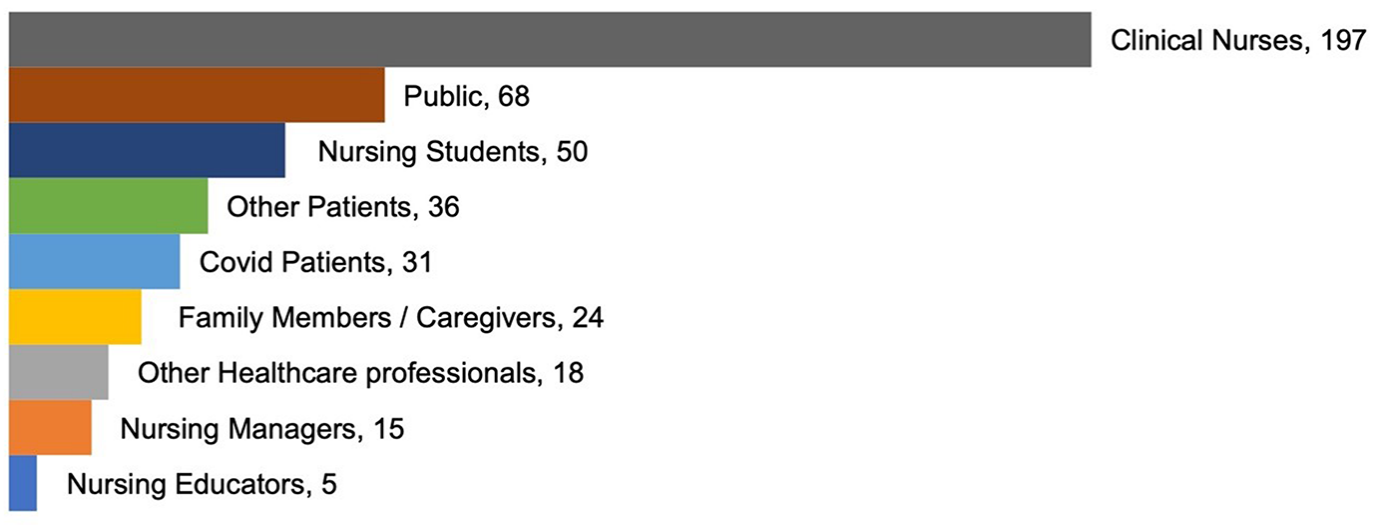
Target population of COVID-19 qualitative nursing research

**Fig. 5:**
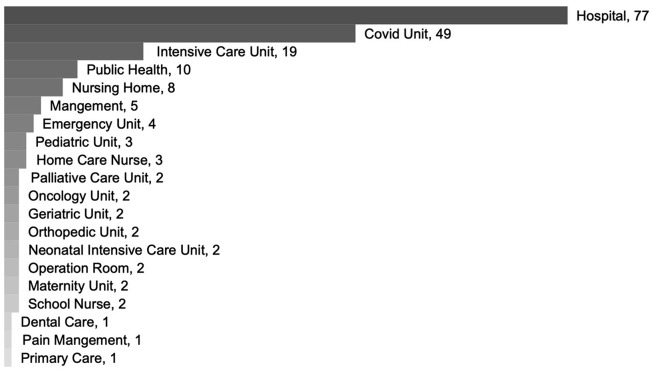
Distribution of clinical nurses

The focuses on COVID-19 qualitative nursing publications were categorized into 7 groups: workforce experience (*n* = 213, 48.0%), pandemic restrictions experience (*n* = 100, 22.5%), learning experience (*n* = 44, 9.9%), infected COVID-19 experience (*n* = 32, 7.2%), hospitalized experience (*n* = 30, 6.8%), psychological perception (*n* = 24, 5.4%), and guideline analysis (*n* = 1, 0.2%) during the COVID-19 pandemic, see Fig. 3. The population was mainly clinical nurses (*n* = 197, 44.4%), nursing managers (*n* = 15, 3.4%), nurse educators (*n* = 5, 1.1%), nursing students (*n* = 50, 11.3%), other healthcare professionals (*n* = 18, 4.1%), COVID-19 patients (*n* = 31, 7.0%), other patients (*n* = 36, 8.1%), family members / caregivers (*n* = 24, 5.4%), and public (*n* = 68, 15.3%). Figures 4 and 5 shows the population distribution of the included articles.

### Methodological quality assessment of COVID-19 qualitative nursing research

Table 3 shows the prevalence of the items for reporting the methodological quality assessment of the included articles.

**Table 3:** Prevalence of the items of reporting the methodological quality assessment

Topics	Items	n	%
Methodological orientation	Approach adopted specified	444	100
	Reporting quality checklist mentioned	135	30.4
Data Collection	Method of data collection mentioned	444	100
	Date of data collection mentioned	365	82.2
	Interviewer/Facilitator	261*	64.9
	Setting of data collection mentioned	327*	81.3
	Interview duration mentioned	340*	84.5
	Audio/Visual recording mentioned	330*	82.0
	Field note mentioned	109*	27.1
	Interview guideline used	351*	87.3
	Interview guideline pre-tested	68*	16.9
Participants	Sample size mentioned	434	97.7
	Sampling methods mentioned	365	82.2
	Description of sample	443	99.9
Data Analysis	Method of data analysis mentioned	415	93.5
	Investigator triangulation mentioned	139	31.3
	Data triangulation mentioned	23	5.2
	Ecological triangulation mentioned	126	28.4
	Source triangulation mentioned	7	1.6
	Using software	167	37.6
	Data saturation mentioned	179	40.3
Presentation of Results	Narrative presentation	444	100
	Quotations presented	401	90.3
	Recurrence of codes presented	21	4.7
	Presentation of graphs	83	18.7
	Participants checking report mentioned	41	9.2

*Note N* = 444, *N = 402 (Articles included using interview / discussion methods)

### Methodological orientation

**Fig. 6:**
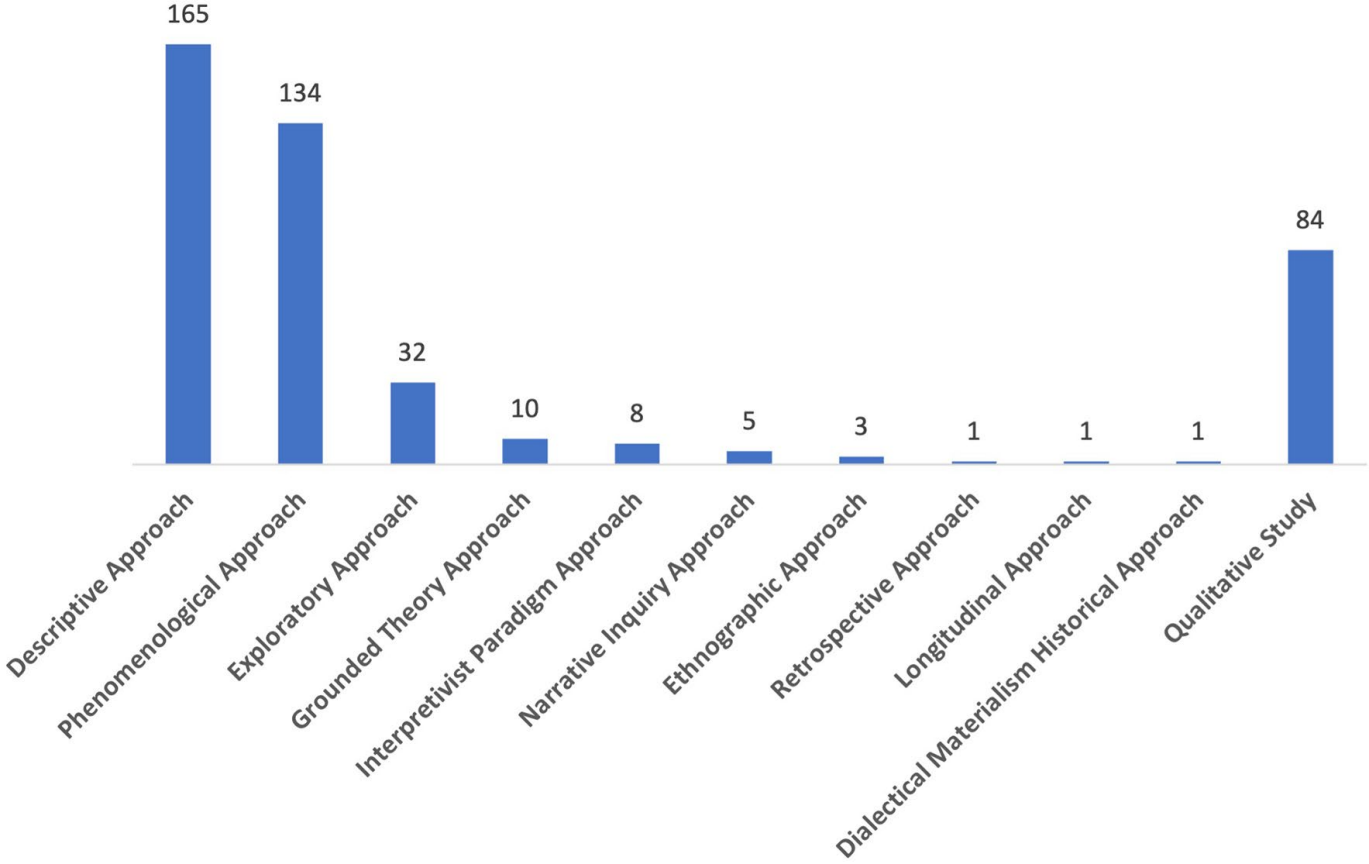
Types of approach adopted

Of the 444 articles, the most adopted approach was the descriptive approach (*n* = 165, 37.1%), Fig. 6 shows the types of approach adopted. Additionally, 84 (18.9%) of the articles only mentioned “qualitative study” without specifying which approach was being adopted.

Only one-third of the analyzed articles (*n* = 135, 30.4%) mentioned employing standardized reporting quality checklists. Among these, the COREQ checklist was the most utilized (*n* = 119, 26.8%), followed by the SRQR checklist (*n* = 16, 3.6%).

### Data collection

Among the articles included, 382 (86%) used interviews/discussions as a data collection method, 20 articles (4.5%) used mixed methods for data collection, and 42 articles (9.5%) that used methods other than interviews/discussions, 2.7% used surveys with open questions, 2.03% carried out document analysis, 1.8% examined diaries, 1.6% analyzed comments on social media, 0.5% used the photovoice method, and finally 0.2% carried out an analysis of audio-newspapers, an analysis of video diaries, an analysis of media interviews, only 1 article used observation as data collection method.

**Fig. 7:**
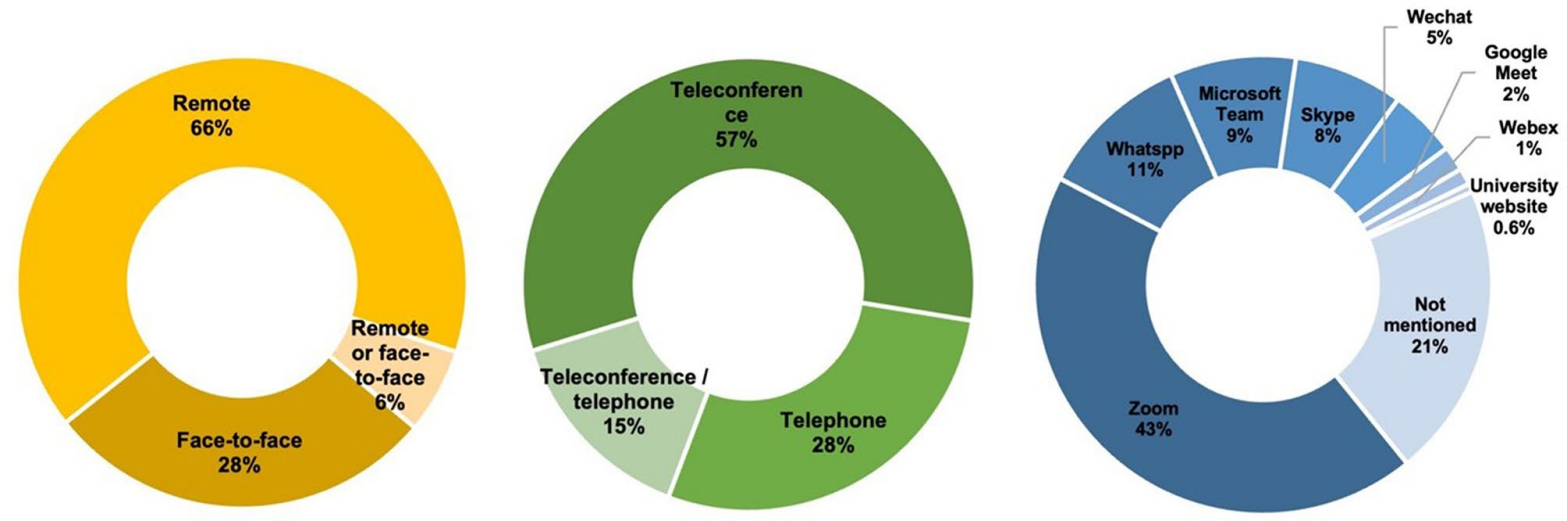
Characteristics of data collection

With the articles using interview/discussion methods, 261 articles (64.9%) specified who conducted the interviews. And 78 of them (19.4%) provided detailed information on their professional profiles. Most articles (*n* = 327, 81.3%) mentioned the setting of data collection, with 65.4% (*n* = 214) conducted remotely, 28.4% (*n* = 93) conducted face-to-face, and 6.1% (*n* = 20) indicated that the interviews were conducted whether remotely or face-to-face depending on participants’ wishes. The remoted interviews were conducted by teleconference (*n* = 134, 57.3%), by telephone (*n* = 66, 28.2%), and by teleconference or telephone (*n* = 34, 14.5%), depending on the choice of participants. The software commonly used for teleconferencing was Zoom (44.5%), WhatsApp (11%), and Microsoft Teams (9.2%), while 35% did not mention which software was used, Fig. 7 shows the characteristics of data collection. Most articles (*n* = 340, 84.8%) specified the duration of the interviews, they were described in two ways: mean duration (*n* = 87, 25.6) or minimum and maximum duration (*n* = 253, 74.4%). Audio recording was most used (86.7%), followed by visual recording (13.0%), and a few (0.3%) mentioned whether audio/visual recording was used. Most articles (*n* = 351, 87.3%) provided interview guidelines, while only 16.9% (*n* = 68) pre-tested them.

### Participants

Most articles (*n* = 434, 97.7%) mentioned the number of samples. Almost all the articles (*n* = 443, 99.9%) provided a detailed description of the samples. The most common sampling method was purposive sampling (*n* = 244, 66.8%), followed by convenience sampling (*n* = 34, 9.3%) and snowball sampling (*n* = 33, 9.0%). Some articles (*n* = 54, 14.8%) used mixed sampling methods.

### Data analysis

**Fig. 8:**
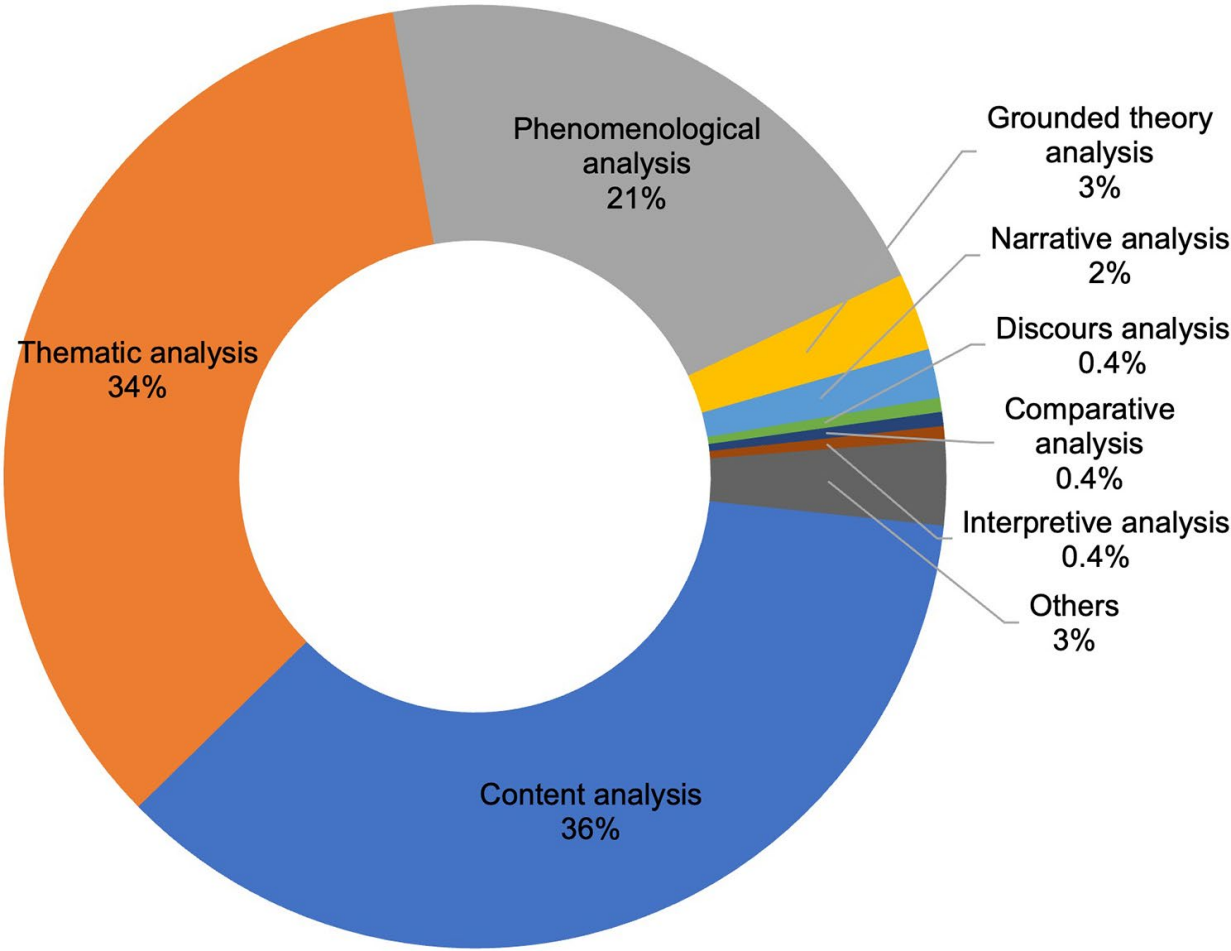
Methods of data analysis

The commonly used methods of analysis were content analysis (*n* = 149, 36.0%) and thematic analysis (*n* = 143, 34.4%) (Fig. 8). And some articles did not specify which method was used (*n* = 14, 3.4%). And the most common software chosen by the authors were NVivo (46.9%), MAXQDA (26.7%), and ATLAS.ti (16.0%).

### Presentation of results

All the articles (100%) presented their results in narrative form. The majority (90.3%) presented quotations in their results. Only 4.7% presented code recurrence. 18.7% used graphics to present their results, and 9.2% mentioned participants checking reports.

## Discussion

This study focused on the identification of the characteristics and reporting quality of qualitative nursing published research related to COVID-19 pandemic. We used a systematic search approach to identify qualitative nursing studies published related to the COVID-19 and then carried out a critical review with the use of content analysis of the identified articles, relying on a checklist created based on two standardized checklists (SRQR and COREQ). A total of 444 published studies were included and critically reviewed. The most productive country was the USA, which corresponds with a bibliometric analysis of COVID-19 research published in a nursing journal. This can be explained by the fact that the USA is one of the most impacted countries by COVID-19 [27] and is one of the most prolific countries regarding nursing research [28]. A significant finding of our study is that the majority of articles were published in journals ranked within Quartile 1. This suggests that the research produced during this period not only addressed urgent topics but also met high academic standards.

In addition, the findings revealed that the most represented topics and target population were related to the workforce experience and clinical nurses respectively, this is consistent with an article that focused on the reflections on nursing research during the pandemic COVID-19 [2]. Interestingly, clinical nurses were the predominant target population of the articles reviewed, this is possibly attributable to the challenge of conducting research with patients and the public due to pandemic-related restrictions. This thematic focus is likely driven by the critical challenge and changes by clinical nurses during the pandemic, highlighting their significant role in the frontline response and the need to understand and support them.

It was surprising to see that the use of standardized checklists to guide research studies by the researchers was notably low, with only 30.4% mentioning the use of standardized checklists. This finding is particularly noteworthy in the context of qualitative nursing research during the COVID-19 pandemic, a period that demanded high-quality evidence to inform rapidly changing clinical practices. The low adoption rate of standardized checklists may reflect gaps in researchers’ awareness or accessibility to these tools, or perhaps a broader issue in the research culture that undervalues structured guidance in study design and reporting, as these checklists aim to improve the quality of reporting these study types and allow readers to better understand the design, conduct, analysis and findings of published studies [25].

Traditional qualitative research data collection methods like interviews and discussions were supposed to be most impacted by the pandemic. Surprisingly, 86% (*n* = 382) of the included articles used interviews or discussions as the data collection methods, and 28% of the researchers remained choosing the face-to-face interview method. We questioned how communication and facial expression were observed if facemasks were worn during the interview. Among the included studies that used the interview method in data collection, a significant proportion with 66% of these interviews were conducted remotely, either by telephone or online. These findings align with the literature reviewed, where nursing researchers in the USA reported an increase in the use of online platforms, as well as sending emails and phone calls for data collection, a trend which has seen a significant increase [8]. Researchers in Japan also reported having to adapt their research methods according to changes in the research environment, moving from in-person interviews to remote telephone interviews, collecting data while maintaining the social distancing, and online data collection [8]. A randomized research study comparing online interviews to in-person interviews person to assess health conditions was conducted in Australia. The results of this study showed that online interviews were preferred by a greater proportion of participants than in-person interviews, and then those assigned to the online group had a lower dropout rate. Additionally, the use of online interviews did not result in a loss of data quality [29]. Another study also indicated that online modalities for conducting qualitative research did not lead to substantially different thematic findings than in-person data collection [30]. These suggest that remote data collection methods would be a good choice for researchers, especially in situations where face-to-face interactions are challenging or not possible. The success of remote interviews in maintaining data quality, participant engagement, and lower dropout rates indicates their viability as a robust alternative to traditional methods. This shift not only ensures the continuity of research during crises like the COVID-19 pandemic but also offers a flexible and efficient approach for future qualitative studies. Embracing remote data collection can enhance the adaptability of research designs and potentially broaden the reach and inclusivity of participant recruitment, making it a valuable methodological option for qualitative nursing researchers.

The adoption of software tools in data analysis was surprisingly low, with only 37% of studies utilizing such resources. This finding suggests a potential area for further development in qualitative research practices, particularly to enhance efficiency and collaboration, especially in scenarios necessitating remote work and data sharing, especially during the pandemic when social contact was limited. In addition, there are other benefits of using qualitative data analysis software, including freedom from manual and administrative tasks, saving time, greater flexibility, and improved validity and reliability, and traceability of qualitative research [31].

In summary, this study carried out an in-depth analysis of data relating to the journals, articles, researchers, and methods used, identifying both strengths and areas requiring improvement. It highlighted the editorial quality of the publications and the methodological diversity observed in qualitative nursing studies linked to the COVID-19 pandemic. We found that many articles demonstrated commendable transparency in explicitly detailing their research approach, data collection processes, sampling methods, and data analysis techniques. However, some areas need improvement. A key aspect is the insufficient representation of strategies to ensure study rigor, such as triangulation and validation by respondents. It is essential to include critical reflection on the role of researchers, potential biases and their influence during the analysis and selection of data for presentation. Additionally, discussions about data saturation and sequential analysis can significantly strengthen the quality of qualitative research reporting. It is important that authors not only explain the methods or techniques they used but also provide clear and detailed justifications for their choices.

The effective translation of nursing research into clinical practice is critical, especially as healthcare professionals heavily depend on the latest research to guide their practices and decisions. The variability in the quality and reliability of research articles can lead to the adoption of clinical practices that may not be supported by strong evidence, potentially affecting patient care and hindering the advancement of nursing practice [32]. Therefore, improving the transparency and rigor of research methodology reporting is essential to ensure that clinical practices are based on reliable and robust evidence. Our study highlights the importance of methodological clarity and the use of standardized checklists in guiding research, This is increasingly relevant as nursing research evolves to meet global health challenges. By ensuring the high quality of reporting qualitative research, we can better bridge the gap between research and clinical practice, leading to improved patient outcomes and more effective healthcare delivery.

### Limitations

It is also essential to recognize that our research method may have some limitations. The diversity of qualitative research methods restricted our assessment to an overview of overall research reporting quality. Additionally, our inclusion criterion based on the first author as a nurse may have excluded studies conducted by nurse-led teams, but where academic conventions led to a different first author. The time limit of the database prevented us from including articles published after January 2023. Finally, we excluded articles not published in English or French, meaning that relevant articles in other languages may have been omitted.

## Conclusions

In conclusion, we urge researchers to provide detailed information in their articles, thereby allowing audiences to carefully evaluate the effectiveness and adequacy of the methods and materials used to produce credible and useful results. We also recommend researchers to adopt validated critical appraisal checklists when conducting their studies. This study highlights the importance of continued reflection on qualitative research practices with a view to improving the reporting quality of future studies in the field of nursing, especially during the special period of a pandemic. Additionally, we plan to compare these results with ancillary studies to assess the characteristics and reporting quality of qualitative nursing research before the COVID-19 pandemic. In the future, we wish to open the way for future studies aimed at exploring the relationships between the different criteria identified and each qualitative approach.

## Data Availability

The data as well as detailed descriptions of the literature search and search outcome (including excluded articles) are available from the corresponding author upon request.

## Abbreviations

COVID-19: Coronavirus disease of 2019
COREQ: Consolidated Criteria for Reporting Qualitative Research
SRQR: Standards for Reporting Qualitative Research
MeSH: Medical Subject Heading
CINAHL: Cumulative Index to Nursing and Allied Health Literature
PRISMA: Preferred Reporting Items for Systematic Reviews and Meta-Analyses
USA: United States of America
